# Ultra-processed foods in public health nutrition: the unanswered questions

**DOI:** 10.1017/S1368980022002105

**Published:** 2023-07

**Authors:** Michael J Gibney

**Affiliations:** Institute of Food and Health, University College Dublin, Belfield, Dublin D04 V1W8, Ireland

## Abstract

There is a growing interest in the study of the degree of food processing and both health and nutritional outcomes. To that end, several definitions of the degree of processing have been proposed. However, when each of these is used on a common database of nutritional, clinical and anthropometric variables, the observed effect of high intakes of highly processed food, varies considerably.. Moreover, assigning a given food by nutritional experts, to its appropriate level of processing, has been shown to be variable. Thus, the subjective definitions of the degree of food processing and the coding of foods according to these classifications is prone to error is prone to error. Another issue that need resolution is the relative importance of the degree of food processing and the formulation of a processed food. Although correlational studies linking processed food and obesity abound, there is a need for more investigative studies.

Since the dawn of nutritional science, foods with a common origin have been categorised into food groups such as milk and milk products, spreadable fats, meat and meat products or vegetables. These categories are usually used to report patterns of food consumption, given that typical food composition tables might contain several thousands of individual foods. There is no record of any ambiguity as to the meaning of such food groups and, using raw data from dietary surveys, the foods within any category can be changed at will, to suit the research question in mind. In recent years, a new categorisation of foods, based on their degree of processing, has been proposed and is a rapidly increasing source of published literature. The present paper seeks to examine the strength of evidence that the degree of food processing is useful in the science of public health nutrition.

## Defining highly processed foods

The most widely used definition of highly processed foods comes from the University of Sao Paulo where the NOVA classification of foods has been developed^(1)^. Within that system, four levels of processing are defined: minimally processed (MP) foods, processed culinary ingredients, processed foods and ultra-processed foods (UPF). The latter are defined as foods which contain *‘substances never or rarely used in kitchens, or classes of additives whose function is to make the final product palatable or more appealing’.* The University of North Carolina (UNC) classification system builds on the NOVA definition of UPF and the European Prospective Investigation in Cancer (EPIC) also developed a definition of highly processed foods^(2,3)^. Finally, the International Food Information Council (IFIC) developed a categorisation of foods based on the degree of processing and two of these categories (ready-to-eat processed foods and prepared meals or foods) are combined to provide a definition of highly processed foods^(4)^. The extent to which these four systems of food processing classification agree on the impact of UPF consumption on biochemical, clinical and anthropometric outcomes was examined using a large Spanish database (PREDIMED-Plus Cohort). Food intake data were recorded for the degree of food processing according to the four approaches (NOVA, UNC, EPIC and IFIC)^(5)^. The results showed that, using a fully adjusted linear model, the interpretation of the relationship between the level of consumption of UPF on the parameters studied varied across the four definitions. The NOVA classification found a positive association between UPF intake and BMI (kg/m^2^) whereas no such association was observed with any other definition of UPF. In contrast, the UNC classification showed an effect of UPF intake on both systolic and diastolic blood pressure but none of the other three definition found this effect. Whereas none of the four classification systems found any link between the level of UPF consumption and LDL-cholesterol levels, three (IFIC, UNC, EPIC) found a positive association between UPF intake and HDL-cholesterol while NOVA alone did not show this association. These contrasting findings, as to the link between UPF intake and measures of health, are due to the subjective approach to defining UPF. There is no scientific basis for choosing any one of these four definitions over the others; however, popular any individual definition might be with authors of papers on highly processed foods and health. Even within a given food processing classification system, there exists subjectivity in assigning individual foods to particular degrees of processing. A French study examined the ability of food and nutrition experts to correctly assign generic or marketed foods to one of the four levels of processing in the NOVA system^(6)^. Irrespective of whether or not the full ingredient data were provided, the authors found a high level of discordance across the evaluators. Clearly, this variability between classification systems coupled with the poor inter-individual assignment of foods to specific processing categories highlights a major problem with the use of food processing classification systems in public health nutrition.

## Homogeneity of ultra-processed food

The NOVA classification of UPF is based on twelve or so food categories (it varies over time) and almost all studies use this broad classification to study the link between intake of UPF and health outcomes^(7)^. Foods defined as UPF generally account for 60 % of total energy intake and, with such a wide coverage of the food chain, it is not surprising that dietary sub-groups can be identified among UPF consumers. The lifestyle prospective study examined the relationship between UPF and the development of type 2 diabetes in over 70 000 adults followed for 41 months^(8)^. Four patterns of UPF consumers were identified using principal component analysis: two involved snacks (one hot, one cold), one represented the traditional Dutch diet and one was high in sweets and pastries. The two snack clusters showed a positive relationship with the onset of diabetes while the sweet and pastry cluster showed a negative relationship. The traditional Dutch diet cluster showed no relationship. Another study found that whereas the totality of UPF categories was associated with all-cause mortality in renal transplant patients, only two of the twelve categories of food within the NOVA definition of UPF (sugar-sweetened beverages and processed meats) showed a significant association with all-cause mortality^(9)^. Both of these studies indicate that the gross classification of twelve food categories into one large category of UPF may yield results which do not correctly drive options within public health nutrition.

## Nutrients or additives

Three studies examined the relationship between the intake of NOVA-defined UPF and chronic disease and have shown that the effect of UPF on chronic disease remained even when the nutritional quality of the diets of individuals was included in the logistic regression analysis^(10–12)^. However, one other study failed to find such an effect^(13)^. This raises the question of whether the true causative agent in UPF is not the nutrient profile but rather, the food additive content. The approval of a food additive for use in the human food chain is subject to very extensive toxicological evaluation in cell lines and with animal models. Carcinogenic and mutagenic properties are included within the toxicological profiles used for approval of a food additive. Subsequent post-approval data from human epidemiological studies may indicate a possible association between an additive and some chronic disease. In that case, the approval for the use of the food additive for human consumption will be re-assessed and either withdrawn, approved for use with altered conditions or the original approval and conditions of use upheld. It is therefore difficult to envisage how a putative significant association between the intake of a food additive and a given chronic disease could be missed in the lengthy and extensive toxicological evaluation of the additive in question. Assessing the occurrence and usage levels of food additive is extremely challenging, putting exposure estimates beyond the abilities of most research groups^(14)^. If sufficient data are available from local or national public analysts’ laboratories on the additive content of commercially produced foods, it is possible to compute exposure data^(15)^. However, given the decades-long consumer concern about food additives, a significant number of food additives currently in use are naturally occurring and estimates of exposure to a given food additive will be confounded by intakes of the chemical in question from natural sources^(16)^. In effect, the only reliable approach is to conduct total diet studies, specifically designed to target food additives. Total diet studies draw on data from food consumption studies but involve the subsequent purchasing and cooking of foods or food groups prior to analysis for a specific food additive content^(17)^. Most large food retailers list the full ingredients of processed foods and it would be valuable as a starting point in studying food additives as causative agents of UPF, to document the occurrence of different additives among high and low consumers of UPF.

## Ultra-processed food and energy balance

Whereas most studies of NOVA-defined UPF intake and obesity have shown positive associations, several key studies have failed to confirm such a link^(7)^. This may be due to what has been previously mentioned, the high level of discordance in coding foods according to the UPF classification system. One randomly controlled study in a metabolic ward setting found that increased consumption of UPF was associated with a rise in body weight while the control arm, fed a MP diet, showed no such trend^(18)^. Because this trial involved *ad libitum* intakes of food, the range of foods offered to either arm of the study at each meal exceeded the predicted energy intake and the two arms of foods offered had identical mean energy densities. However, from the foods offered, those chosen by the subjects in the UPF arm had a higher energy density than the foods chosen on the MP diet. Energy density is a known driver of energy intake and thus should be controlled for in studies of UPF intake and obesity. Sadly, it has never been retained as a variable in any of the studies of UPF intake and obesity. A Dutch study has shown that whereas the mean energy density of UPF is higher that less processed foods, the within-category variability is also very high^(19)^. Thus, MP foods can have a high energy density (avocados, peanuts, butter) while foods designated as highly processed can have a low energy density (breads, breakfast cereals, flavoured low-fat yogurts).

In the Randomised Controlled Trial that found an association between the intake of UPF and obesity, a higher eating rate was also found on the UPF arm of the trial. A high eating rate is strongly associated with weight gain^(20)^. To differentiate between the effects of degree of processing and eating rate, a recent study compared the eating rates of hard and soft lunches representative of both minimally and UPF^(21)^. The hard, MP and hard-ultra-processed meals were consumed at an equally slower rate than their soft counterparts (UPF and MP), reducing weight of food intake by 21 % and energy intake by 26 %, irrespective of the degree of processing. Texture alone significantly influenced eating rate. Thus, more studies on the physical nature of processed foods are needed to fully understand any putative effect of UPF on food choice.

## Conclusions

In any scientific discipline, definitions must be objectively derived. The difficulty with the present approach is that all definitions are subjective, simply reflecting the personal opinions of those deriving the definition. The NOVA definition of UPF refers to…. ‘*additives whose function is to make the final product palatable or more appealing’.* Palatability is not just a function of a given food but is primarily determined by genetic, phenotypic and environmental factors^(22)^. Palatability is therefore a subjective term as in ‘*chacun à son gout*’ (each to their own taste). The subjective nature of the definition continues with reference to the ability of food additives to make a food ‘more appealing’. If a preservative is used in a bread, does that make it ‘more appealing’ to a consumer than an identical bread with no additives present? If the degree and nature of processing of foods are to be considered as an important driver of public health nutrition, then some level of objectivity in the definition of highly processed foods is needed. Moreover, the selection of candidate foods for consideration as highly processed must first be examined to understand their population impact on nutrient intakes. Breads and breakfast cereals are classed as NOVA UPF foods and yet they make a considerable positive impact on population nutrient intake. Further gains in nutrient intake with bread and breakfast cereals can be achieved by promoting whole grain varieties and through reformulation. However, the NOVA recommendation that all UPF foods be avoided, including industrially prepared breads and breakfast cereals, does not make sense for public health nutrition policy. Moreover, NOVA opposes the concept of reformulation of foods on the grounds that one cannot make an unhealthy food (subjectively defined) healthy^(23,24)^. This ignores extensive efforts by governments to encourage reformulation of foods to lower salt, added sugars and fats. By the same token, foods that are considered treats, such as chocolate, and which make a modest contribution to energy intake, might be excluded from consideration as UPF. Presently chocolate is deemed to be UPF and thus to be avoided. If the degree and nature of food processing are to be considered within the strategies of public health nutrition policies, a robust, objective, evidence-based definition must be devised and the criteria for considering a food as highly processed must first take account of that food’s impact on population nutrient intake.
